# Impact of Destination Image Formation on Tourist Trust: Mediating Role of Tourist Satisfaction

**DOI:** 10.3389/fpsyg.2022.845538

**Published:** 2022-04-01

**Authors:** Abdelhamid Jebbouri, Heqing Zhang, Zahid Imran, Javed Iqbal, Nasser Bouchiba

**Affiliations:** ^1^Department of Tourism, School of Management, Guangzhou University, Guangzhou, China; ^2^Lahore Business School, University of Lahore, Lahore, Pakistan; ^3^School of Education, Guangzhou University, Guangzhou, China; ^4^Department of Political Science, Sun Yat-sen University, Guangzhou, China

**Keywords:** destination image formation, tourist satisfaction, tourist trust, authenticity, structural equation modeling, measurement analysis

## Abstract

Tourist destinations with cultural heritage have arisen as a prominent issue in tourism literature. Creating a positive image of the destination can influence tourists’ satisfaction and willingness to return. The goal of this research is to investigate the relationship between destination image formation (DIF), tourist satisfaction (TS), and tourist trust (TT). As a result, the structural relationships between local community participation (LCP), authenticity (A), access to local products (ALP), TS, and TT were investigated in this study. This study used a quantitative approach based on a survey of 644 domestic and foreign tourists visiting the Guangdong cities of Guangzhou, Foshan, and Shenzhen. The statistical software SmartPLS 3.3.3 was used to determine the relationship between variables in the research model using structural equation modeling. The outcomes show a positive correlation between LCP, A, and ALP, which led to tourist satisfaction and, eventually, tourist trust. It is concluded that the DIF and TS may result in increased tourist trust. There is also a discussion of additional theoretical contributions, practical implications, and limitations. The outcomes of this study will help to shed light on the variables that encourage and promote tourism in developing countries.

## Introduction

Today, tourism is among the most vital industries on the planet (Roodurmun and Juwaheer, 2010; Som and Badarneh, 2011). It is a major source of revenue and a growth driver for other industries like retail, transportation, and infrastructure. Recent years have seen a surge in tourism development, particularly in underdeveloped countries where governments have taken proactive measures (Bansbardi et al., 2013). Numerous research has been conducted in this area to determine the economic effect of tourism in various countries (Baloglu and McCleary, 1999; Kozak et al., 2007). Moreira and Iao assert that there is fierce competition among tourist destinations worldwide for tourists. Due to the tourism industry’s intense competition, building and maintaining positive images of destinations is necessary. Destination images are crucial in determining behavior or travel behavior in terms of destination selection, desire to revisit, and intention to spread word of mouth (Onatski et al., 2014; Webster and Ivanov, 2014).

The tourism sector has become a key contributor to the Chinese economy since starting the reforms and opening in the early 1800s (Williams et al., 2016). The boom in the tourism industry was and has been contributed by the emergence of affluent middle-class people as well as the easing of restrictions on movement for both locals and foreign visitors. Besides, the sector has expanded to become one of the globe’s most-watched outbound and inbound tourist markets (Thomala, 2020). The number of local trips in China reached around 2.4 billion in 2020, although the year has been characterized by the COVID 19 pandemic (Wang K. et al., 2020). This would indicate an increase of more than 50% compared to the trips made within a decade ago. As of 2016, total Chinese revenue on tourism and travel amounted to 3.94 trillion yuan, translating to a 15.2% increase from the previous year (Li et al., 2016). The industry improved China’s gross domestic product by 2.1% and an estimated 22.5 million jobs (Thomala, 2020; Wang K. et al., 2020). All these have been contributed by considering aspects such as the multiple heritage sites, infrastructure, community interactions, and tourists’ safety, among other factors such as the destination image formation (DIF) and tourist satisfaction (TS).

The term “destination image” refers to the process through which people try to form an impression of a particular vacation destination. The destination image encompasses beliefs, feelings, perceptions, and knowledge about a destination, in addition to direct and indirect information gained while traveling to the destination, such as through associations, tourism-related channels, social platforms, and the Internet facility. The significance of developing destination images for managers stems from the fact that they have a considerable impact on tourists’ purchasing decisions and actions (Pike and Ryan, 2004). Further, studies have shown how people are more drawn to a destination’s image based on their emotions and impressions rather than the facts of the destination itself (Kim and Yoon, 2003; Abodeeb et al., 2015). Kozak (2001) investigated how tourists’ perceptions of a destination are developed, both domestically and internationally. According to Kozak, tourists who are delighted with their vacations are eager to tell their friends and family about it. When tourists are happy, they are more apt to return to the same destination and suggest it to others. The overall image of a destination is an essential mediator in the relationship between brand associations and future visitor behavior, according to Qu et al. (2011). Furthermore, Hanzaee and Saeedi (2011) found that the overall image plays an important role in helping to mediate the association between destination brand associations and future tourist behavior.

The effect of DIF and TS on TT has been a popular subject of tourist studies. When making strategic marketing decisions for tourism locations, it is crucial to determine the destination’s image due to the assumption that a positive DIF creates trust toward tourist destinations and increases tourists’ satisfaction (Coban, 2012). Trust is a notion deeply linked to tourist satisfaction; consequently, regardless of the concepts, trustful tourists report a high level of fulfillment. TT is reliant upon TS (i.e., attractions, lodging, accessibility, facilities, and activities) and fulfillment of expectations (Chi, 2012). The overall image of the destination (attractions, accommodation, accessibility, amenities, activities, local community, and commerce) is a predictor of TT (Prayag, 2009). TT is impacted by essential services, attractions, and accessibilities (Kastenholz et al., 2013). TT in a destination is impacted by the destination’s image, personal involvement, place attachment, and overall satisfaction (Prayag, 2009).

Three conceptual tourism destination research gaps will be addressed in the present study. (a) It will first look into the individual link between DIF and TT. (b) It will investigate the connection between DIF and TS. (c) It will look at the relationship between TS and TT. (d) It will examine the role of TS as a mediator between DIF and TT. Previously, very few studies have explored such kinds of relationships. This is the first study investigating how destination image formation, directly and indirectly, affects tourist trust in the Chinese heritage tourism context.

An integrated model is presented in this study, which adds to the existing body of tourist literature by providing a fresh perspective about the role of DIF in affecting the TS that leads to TT. The findings can help those in charge of marketing tourist destinations in designing and implementing market-driven approaches that promote consumer satisfaction and trust through strategies that focus on destination image formation represented in Authenticity, LCP, and ALP.

The absence of actual studies involving Asian tourists is the second conceptual research gap. It is anticipated that Asia will become the globe’s most popular vacation destination and largest tourist-producing region in the near future. Notably, Tourism in PRC was almost non-existent prior to 1978, when it unlocked its gates to the outside world. Since then, China has become a significant tourism marketplace. China’s tourism officials have been concentrating their efforts on evolving national tourism (Zhang et al., 2016), and this study examines domestic Chinese tourists primarily.

In addition to addressing the first two gaps, this study addresses the third gap, which is the need for extended research into visitors’ perceptions and behavior regarding their experiences in Heritage Sites (Su et al., 2020), Particularly those who are based in China. Numerous scholarly studies (e.g., Landorf, 2009; Hosseini et al., 2021; Yang et al., 2021) have concentrated on the sustainability and resource management of Heritage Sites, additionally to the significance of Heritage Sites in attracting international tourists (Su and Li, 2012). There has been a relatively slight investigation into the impact of tourist destination image formation (LCP, A, ALP), on tourist trust, by using tourist satisfaction as a mediated variable in heritage sites, particularly those in China. For this purpose, we developed a synthesized research framework to explore the following research questions:

**RQ1:** How does DIF and tourist satisfaction influence tourist trust?
**RQ2:** How do DIF influences tourist satisfaction impact on tourist trust?
**RQ3:** How does tourist satisfaction mediate the relationship between DIF and tourist trust?

This paper’s structure is as follows: section “Review of literature” includes a literature review. Second, section “Conceptual Framework” covers how the hypotheses were generated, while section “Research Methodology” explains the research methodology and the statistical analysis of this study, respectively. Section “Discussion” contains the discussion and closing remarks. The study comes to a close with a review of the study’s limitations as well as future research prospects.

## Review of Literature

### Destination Image Formation

Destination image formation is defined as the collection of an individual’s beliefs and thoughts about a specific environment or setting. In the same vein, the environmental and social aspects of a destination’s image quality are two of the most important factors in determining its perceived quality (Jiang et al., 2016). Previous studies (e.g., Soliman, 2019; Carvalho, 2022; Dai et al., 2022) have shown that the image of a location is a significant component that impacts travelers’ decision-making, destination selection, and future behavior. Image has been a well-known term in the disciplines of customer behavior and marketing for a long time (Stepchenkova and Morrison, 2008). According to Del Bosque et al. (2006), image results from customers’ perception of the company, consisting of impressions, beliefs, and feelings toward a company. Conversely, DIF also includes the participation of the local community (LCP), authenticity (A), access to local products (ALP), and the services provided to tourists. In addition, destination image has been widely recognized as an important construct that influences tourist behavior, tourism-related decisions, and destination marketing (Picazo and Moreno-Gil, 2019; Song et al., 2019). Researchers generally agree that destination image include several components, such as cognitive, affective, and conative elements (Gartner, 1994). Semantic designative, evaluative, and prescriptive aspects have also been identified. Elbaz et al. (2021) identified four dimensions of destination attractiveness, namely access, amenities, scenery and locals, while Jiang et al. (2016) identified destination service infrastructure and destination facilities as the most significant factors of a tourism destination for international visitors. Travelers are increasingly relying on information from a wide range of online resources, thanks to the rise of the internet. However, there may be significant visual disparities and inconsistencies between various sources, platforms, or agents (Choi et al., 2007; Tang and Jang, 2009). Despite the broad theoretical consensus in favor of LCP in tourism strategies, in practice, destination guests rarely have real influence over important choices (Dragouni and Fouseki, 2018; Su et al., 2019). Tourist visits in China have been affected by the authenticity and commodification of local community objects and local community practices. Cultural heritage is a priceless asset that contributes to the cultural and economic qualities of the host community in a tourist destination (Yang et al., 2017). Intangible cultural heritage with the community is protected to preserve its uniqueness and vulnerability. The protection of the cultural heritage is done through school education, folk protection, salvage operation, and museum preservation.

### Tourist Satisfaction

In the tourism industry, a great deal of research has been conducted on customer satisfaction (Ramkissoon and Mavondo, 2015). The pleasure a customer experiences after a purchase or a series of customer-product interactions is called satisfaction. According to the literature, visitors’ perceptions of their travel experience begin with the mental assessment of travelers’ encounters with different destination characteristics (Sharma and Nayak, 2019). Tourist satisfaction with natural and cultural sites could increase their attachment to a location (Ramkissoon, 2016). Therefore, Organizations responsible for destination management (DMOs) are more concerned with providing and maintaining the characteristics appropriately for travelers to enhance the quality of their vacations and make them memorable (Jing and Rashid, 2018). Additionally, consistent execution of destination characteristics might attract additional tourists, ensuring their satisfaction (Sangpikul, 2018). Formerly, several scholars have identified several destination characteristics that can be broadly classified as commodities or services. For example, Lo et al. (2013) divided destination characteristics into four categories: cultural/heritage, social, economic, and environmental. According to Sevinç and Güzel (2017), various characteristics serve as attracting and satisfying elements for tourists. Sukiman et al. (2013) established a link between both visitor satisfaction and travel experience. In addition, Ragavan et al. (2014) and Valduga et al. (2019) assessed tourists’ perceptions and satisfaction with a tourist destination by evaluating a variety of destination attributes such as accommodation, food, attractions, image, products, accessibility, culture, communities, and price, as well as other attractions that are important components of a tourist destination. Similarly, Ghosh and Sofique (2012) stated that cultural/patrimonial attractions are essential to the tourism product. Tasci and Boylu (2010), on the other hand, focused on the issue of safety during tourists’ trips to measure their satisfaction. In support, Manui and Wongsai (2017) conducted research on international tourists’ feelings of safety while visiting the island. In addition, furthermore, Kim et al. (2015) listed the essential criteria of tourist-friendly destinations. As a result, measure their satisfaction. Accommodation, accessibility, attractions, restaurants, and safety are among the key features.

### Tourist Trust

Trust is defined in tourism literature as the dependability and credibility of important factors associated with tourist destinations (Marinao et al., 2012; Artigas et al., 2017). It is critical for the development of tourism and societal well-being (Ramkissoon, 2020). Trust is a focal point of tourists’ experience, enabling marketers to understand and optimize satisfaction and performance across destinations (Elbaz et al., 2021). A visitor’s willingness to put their faith in a destination’s capability to meet their desires is a key component in building trust, according to Abubakar and Ilkan (2016). Trust underpins a number of critical travel decision-making components, including visitor satisfaction, willingness to return, commitment, and loyalty (Beutler et al., 2011). A destination that instills trust in the eyes and minds of tourists can simply be marketed (Abubakar, 2016).

Previous research has confirmed that TT has an effect on tourists’ risk perception and emotional bonding to a site (Kim et al., 2009; Chen and Phou, 2013), and inspected the relationship between individuals’ trust, their intentions, and their conduct in a variety of circumstances (Chen et al., 2011; Hassan and Soliman, 2021; Ramkissoon, 2020). As well as their emotional connection to a particular destination (Liu J. et al., 2019). Travelers’ intention is affected by a number of factors, including trust (Hassan and Soliman, 2021).

Tourists are more inclined to pay a visit to places they think to be reliable and reputable. In the long run, tourists may form a personal connection with trustworthy places (Chen and Phou, 2013). Travelers may be inclined to choose a well-known place as a means of reducing the risk of travel (Choi et al., 2016). By meeting tourists’ emotional and basic requirements, destinations and tourists can build mutual trust (Roodurmun and Juwaheer, 2010). According to the findings of a recent study, some tourist locations have been able to acquire the trust of their visitors because of their open pricing policies and attention to detail in their landscaping. Additionally, social media reviews revealed that effective traffic management and tourist-friendly amenities were critical in building visitors’ trust in a destination (Liu J. et al., 2019). The existing literature does not have a standard scale for the assessment of TT in a destination. The majority of tourist research has used a trust instrument borrowed from other fields, such as social science (Liu J. et al., 2019), branding (Delgado-Ballester, 2004), and e-commerce (Liu J. et al., 2019). Certain researchers (Marinao et al., 2012; Artigas et al., 2017) established a scale based on a qualitative approach without requiring formal attestations.

## Conceptual Framework

The current study highlights how destination image formation positively and significantly influences the tourist trust using tourist satisfaction as mediating variables. Previously Destination Image Formation (LCP, A, and ALP), TS, and Tourist Trust have significant research attention (Endah et al., 2017; Yun et al., 2019). The theory of the socio-cultural approach in tourism contends that DIF may play an important role in the tourist trust (Endah et al., 2017). The conceptual framework of the research is divided into four main parts. First, it investigates the individual relationship between DIF and TT; secondly, we also inspect the relationship between DIF and TS. Third, we investigate the relationship between TS and TT. The fourth and last part of this study tests the mediating relationship of TS between DIF and TT. Therefore, in support of the above relationships, many scholars provide support in previous studies.

Several studies indicated that DIF (LCP, A, and ALP) positively influence TT preferences (Dragouni and Fouseki, 2018). Similarly, a number of studies revealed that destination image formation (LCP, A, and ALP) positively influence tourist satisfaction (Engeset and Elvekrok, 2015; Ridho et al., 2021). Moreover, previous studies explained that tourist satisfaction positively influences tourist trust (Kim et al., 2017; Al-Ansi et al., 2019). Furthermore, TS mediates between DIF and TT (Endah et al., 2017; Jeong and Kim, 2019a). To do so, it empirically analyzes these relationships and highlights the influence of DIF on TT through TS. The study also contributes to the clarification of the previous literature by addressing the role of DIF in affecting the TS that leads to TT.

**FIGURE 1:**
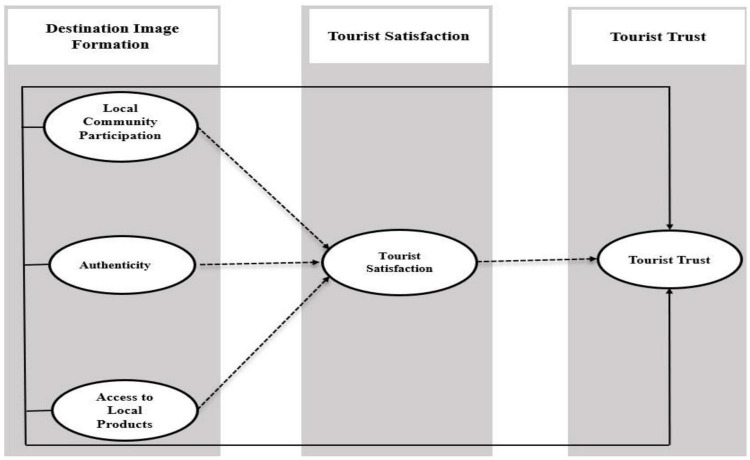
Conceptual framework.

In addition, it is widely acknowledged that the tourists can attain a better image through receiving destination image better and getting more satisfaction, especially in emerging nations like China. While TT is defined broadly as the confidence, a visitor develops in a product or service provider during a visit to a tourist destination due to a relationship exchange between two parties (Sirdeshmukh et al., 2002). They could also be examined in relation to the DIF of tourists. Finally, it has been found that DIF can help TS to improve TT (Guzman-Parra et al., 2016; Jeong and Kim, 2019b). Based on the above-discussed literature, we draw below mentioned conceptual framework (see Figure 1).

### Hypotheses Designing

#### Destination Image Formation and Tourist Trust

Similarly, Wallin Andreassen and Lindestad (1998) defined image as the accumulation of experience gained by purchasing or consuming time. Further, a company’s image is shaped by its advertising and public relations, along with word-of-mouth and the personal experiences that customers have when using the products and services that the firm provides at tourist destinations (Nguyen and Leblanc, 2001). The hotel and tourist industry rely on greater relationships among its stakeholders (Kim and Kim, 2019). As an example, the hotel’s employees should provide its guests complimentary room upgrades and unique attention in order to build their trust and faith in the establishment. When it comes to tourist happiness and loyalty to a destination, trust is an essential factor (Artigas et al., 2017). In today’s competitive market, retaining tourist trust is the most important problem for service businesses (Huang and Su, 2010). According to Lee et al. (2011), the emotion of loyalty or fondness for a specific service, product, or destination can be defined as trust.

Most studies explored that destination image formation in services firms found that image influences consumer trust and loyalty (Chien-Hsiung, 2011; Malik et al., 2012). Then Davies and Chun (2002) specified that destination image is one determinant of tourist trust. Further, this study revealed that destination image directly relates to tourist trust—similarly, Kim (2014) found that destination image influences tourist intentions to come back or revisit. Another study revealed that local community participation increased tourist trust through institutional credibility, which significantly influenced both individual and collective preferences (Dragouni and Fouseki, 2018).

Furthermore, another study explored the significant role of authenticity in formulating TT. The empirical results indicated the importance of online reviews, perceived authenticity, and trust in the tourism framework (Jeong et al., 2019). Several studies discussed the role of access to local products and building trust and personal relationships among tourism stakeholders. The results revealed the positive role of access to local products to enhance tourist trust (Roy et al., 2017). So, one could assert that the destination must be able to create a positive service image in tourists’ thoughts as it can affect tourist trust at a specific destination. Similarly, the studies found that the destination’s image influences tourists’ perceived value (Sadeh et al., 2012). As a result of the preceding argument, the following hypotheses were formulated to measure the relationships:

**H1.1**: LCP has a beneficial and significant impact on TT.
**H1.2**: A has a positive and significant impact on TT.
**H1.3**: ALP has a positive and significant impact on TT.

#### Destination Image Formation and Tourist Satisfaction

Tourist satisfaction refers to a tourist’s emotional or affective assessment of a tourist destination’s product or service utility. Alternatively, it establishes a sense of comfort, delight, and acceptance for the products or services being used or consumed (Oliver, 1999). Previous research has emphasized the significant function of satisfaction because of its ability to accurately predict what a traveler or tourist will want in the future (Jani and Han, 2014b). It has become increasingly important for managers and marketers in international tourist destinations to ensure that their visitors have a positive and memorable experience (Lee S. et al., 2017). According to Mai et al. (2019), TS is an emotional response that occurs when comparing expectations and perceptions of service performance to real perceptions gained via physical encounters with products or services. Tourist satisfaction has long been a top priority for many countries, especially emerging ones, in the quest to grow their tourism industries (i.e., China).

Several studies explored that DIF (LCP, A, and ALP) influences tourists’ perceived value and tourist satisfaction (Jamal et al., 2011; Sadeh et al., 2012; Rajesh, 2013). Similarly, destination image formation affects tourists’ behavioral intentions through TS (Chen and Tsai, 2007; Mohamad et al., 2011). According to Kandampully (2000) and Yeoman and McMahon-Beatte (2016), tourism is a collection of natural-related products and services that are primarily linked with a destination. A tourism destination is not a stand-alone product but rather the result of a mix of numerous features of tourism destinations that tourists consider when deciding whether to visit or revisit (Framke, 2002). Tourism destination formation, according to Della Corte et al. (2015), has a beneficial effect on tourist services. Other facilities could help travelers retain information and foster good conversation about the destination among customers. Similarly, Ridho et al. (2021) have discussed the significant role of local community participation in enhancing tourist satisfaction. Engeset and Elvekrok (2015) explored that authenticity effectively increases tourist satisfaction among tourists. Alegre and Garau (2011) discussed that access to local products significantly impacts tourist satisfaction. Based on this discussion, it was predicted that DIF, A, and ALP has a positive influence on TS, and the following hypotheses were formulated to measure these relationships:

**H2.1**: LCP has a positive and significant impact on TS.
**H2.2**: A has a positive and significant impact on TS.
**H2.3**: ALP has a positive and significant impact on TS.

#### Tourist Satisfaction and Tourist Trust

Previous research has highlighted the importance of satisfaction due to its remarkable accuracy in predicting a traveler’s or tourist’s future aspiration (Jani and Han, 2014a). According to Taie (2013), an increase in satisfaction can improve tourists’ loyalty through the mediating role of trust. Satisfaction and loyalty are critical components; It is essential to know the psychology of travelers in leisure, hospitality, and tourism in order for a location to be successful. Specifically, a customer who values loyalty is dedicated to consistently repurchasing or patronizing a company or product in the future (Elbaz et al., 2021). International tourism destinations’ marketers and managers are increasingly focused on increasing visitor satisfaction and creating an unforgettable travel experience (Lee C.-F. et al., 2017). Hassan and Soliman (2021) illustrated that trust positively impacted holidaymakers’ intention to revisit the destination. Moreover, some prior studies have investigated the connections among satisfaction, trust, and loyalty. For instance, dos Santos and Basso (2012) discern that the tourist-healthcare provider relationship moderately impacts intent formation, while trust is developed in both personnel and the enterprise based on tourists’ satisfaction with grievance handling systems. In turn, positive word-of-mouth and repurchase intention are fundamentally triggered by trust. There has recently been much discussion about Muslim travelers’ satisfaction with the destination, and there has been an apparent desire for halal travel. Muslim tourist satisfaction is generated, according to Olya and Al-Ansi (2018), by the variety and quality of halal products and services available at the tourist attraction, which comprises medical, environmental, spiritual, and quality performances. A visitor’s confidence and certainty toward product or service providers at tourism sites/places due to a relationship exchange between the two parties is commonly defined as tourist trust (Sirdeshmukh et al., 2002). The hospitality and tourism industries rely on developing and sustaining relationships among their stakeholders (Kim et al., 2011). TS and loyalty to a tourist destination are closely tied to their level of trust in the place they visit (Artigas et al., 2017). This study assessed the tourist satisfaction influence on tourist trust, and the following hypothesis was made for this purpose:

**H3:** TS has a positive and significant impact on TT.

#### Mediating Role of Tourist Satisfaction

According to Davies and Chun (2002), DIF is one determinant of TS and trust. Additionally, this study also discovered that destination image is directly related to satisfaction, and satisfaction directly affects trust. Bigne et al. (2001) state that image influences three components at the same time: the perception of how good or bad a product or service is, how satisfied the person is with it, and how trustworthy they are. Another study looked into the role of services in forming an international destination’s image, such as health quality, psychological, and environmental issues, all of which influence TS and trust (Liu X. et al., 2019). Similarly, the findings of Endah et al. (2017) shed light on the relationship between DIF and satisfaction toward trust and behavioral intention. We found fewer studies that explored the mediating role of TS in the relationship between DIF and TT. Therefore, the current study explored the mediating role of TS in the relationship between DIF and TT.

**H4.1**: TS mediates the relationship between LCP and TT.
**H4.2**: TS mediates the relationship between A and TT.
**H4.3:** TS mediates the relationship between ALP and TT.

## Research Methodology

This research has been conducted in the tourism sector of China as an emerging nation. Currently, several studies have been concentrated on destination image formation, tourists’ satisfaction, and tourist trust in advanced countries such as America, Austria, Spain, South Korea, and France, etc. (Guzman-Parra et al., 2016; Wu et al., 2021), with comparatively fewer studies have been conducted in emerging nations which is increased amputates of the scholars to get more diverse corporate viewpoints. Our rationale for doing this study is to establish a link between LCP, A, ALP, and DIF, all of which contribute to TS and, ultimately, TT. Thus, the Chinese tourism industry is ideal for this study since it is easy to access for the researchers. A survey was identified as the best method for gathering data for this study, and a questionnaire was designed to that end.

### Research Approach

For the empirical testing of hypotheses, a deductive technique was used in conjunction with a positivist philosophical paradigm. We found the fundamental patterns and correlations between variables, and we generalized our findings. The results of the testing of hypotheses contribute to the relevant realm of knowledge. To minimize bias, positivism was chosen, as it examined the sole external reality objectively. To get a representative sample from a broad population, a survey was undertaken (Beutler et al., 2011). The respondents rated the survey as reliable, and the resulting data was easily standardized and compared (Sekaran, 2006). Due to budget constraints, a cross-sectional research strategy was proposed. The data were gathered using a seven-point Likert scale.

### Development of Instruments

This study used destination image formation dimensions (LCP, A, and ALP) as independent variables, tourist trust as the dependent variable, and TS as a mediator. The study used three characteristics of DIF as independent variables (LCP, A, and ALP), TT as a dependent variable, and TS as a mediator. The questionnaire’s initial section described the study’s purpose and included instructions. Additionally, this section included anonymity and privacy disclaimers and inquired about respondents’ personal information, such as gender, age, income level, and field of education. The second part described the items associated with DIF (15 items), TS (7 items), and TT variables (6 items).

All assertions were evaluated using a seven-point Likert scale (from 1 to 7: strongly disagree to strongly agree). Before collecting the final data, the questionnaire items were translated into Chinese using the back-translation method. A pilot study of 30 participants was conducted to determine the questionnaire’s reliability and validity. All participants in the pilot study shared similar demographic characteristics with the primary study sample, allowing for the trial of final data analysis. The pilot study participants were aware of the study’s purpose, and they gave some suggestions for minor changes to the questionnaire. We made modifications in response to their feedback to ensure that all participants comprehended all items and could effectively complete the questionnaires. The amended questionnaire was used to obtain the final data.

### Variables Measurements

#### Local Community Participation

The LCP-related Items were adapted from a variety of sources. Gursoy et al. (2010): this section included four items, each using a 7-point Likert-type scale, ranging from 1 (strongly disagree) to 7 (strongly agree). The sample of the items related to local community participation included as “I feel at home in this community,” “I have an interest in knowing what goes on in this community,” and “I regret moving away from this community.” The threshold value of Cronbach’s alpha is 0.70. The local community participation Cronbach’s alpha was 0.775; thus, the instrument is appropriate to collect the final data (see Table 1).

**TABLE 1:** Reliability and validity.

Sub-scales	Factor loading	Cronbach’s alpha	Rho_A	Composite reliability	Average variance extracted (AVE)
**Local community participation**		0.775	0.777	0.856	0.599
LCP1	0.774				
LCP2	0.805				
LCP3	0.804				
LCP4	0.708				
**Authenticity**		0.862	0.863	0.901	0.645
A1	0.785				
A2	0.836				
A3	0.800				
A4	0.816				
A5	0.778				
**Access to local product**		0.891	0.893	0.917	0.648
ALP1	0.814				
ALP2	0.851				
ALP3	0.795				
ALP4	0.770				
ALP5	0.765				
ALP6	0.831				
**Tourist satisfaction**		0.890	0.898	0.916	0.610
TS1	0.610				
TS2	0.805				
TS3	0.814				
TS4	0.806				
TS5	0.827				
TS6	0.801				
TS7	0.781				
**Tourist trust**		0.815	0.821	0.867	0.523
TT1	0.779				
TT2	0.798				
TT3	0.668				
TT4	0.697				
TT5	0.624				
TT6	0.755				

#### Authenticity

The authenticity-related items were altered from Lee et al. (2020). This section comprised five items, with each item using a 7-point Likert-type scale, ranging from 1 (strongly disagree) to 7 (strongly agree). The sample of the items related to authenticity included as “I like the way the city blends with attractive landscape and historical ensemble, which offer many interesting places to visit,” and “In this place, I can experience the traditional Chinese lifestyle.” The threshold value of Cronbach’s alpha is 0.70. The authenticity Cronbach’s alpha was 0.862; thus, the instrument is suitable to collect the final data (see Table 1).

#### Access to Local Products

The six ALP-related items were altered from Puciato et al. (2020), with each item using a 7-point Likert-type scale, ranging from 1 (strongly disagree) to 7 (strongly agree). The sample of the items related to access to local products included as “My process of selecting this location as a destination was based on objective premises,” and “I collected information before selecting this place as a heritage destination (e.g., service quality, shop atmosphere, gift prices).” The threshold value of Cronbach’s alpha is 0.70. The access to local products Cronbach’s alpha was 0.891; thus, the instrument is appropriate to collect the final data (see Table 1).

#### Tourist Satisfaction

The TS-related items were adapted from Aliman et al. (2014) and Biswas et al. (2021). This section comprised seven items, with each item using a 7-point Likert-type scale, ranging from 1 (strongly disagree) to 7 (strongly agree). The sample of the items related to TS as “I am happy with my decision to visit this place,” “I have a lot of interests in visiting tourist destinations in China,” and “This place has a good standard of hygiene and cleanliness.” The threshold value of Cronbach’s alpha is 0.70. The TS Cronbach’s alpha was 0.890; thus, the instrument is appropriate to collect the final data (see Table 1).

#### Tourist Trust

The TT-related items were altered from Liu J. et al. (2019). This section comprised six items, with each item using a 7-point Likert-type scale, ranging from 1 (strongly disagree) to 7 (strongly agree). The sample of the items related Tourist Trust as “The inhabitants of this place are honest” and “The institutions of this place do their work well.” The threshold value of Cronbach’s alpha is 0.70. The TT Cronbach’s alpha was 0.815; thus, the instrument is appropriate to collect the final data (see Table 1).

### Sampling and Data Collection

Guangdong province in China was the location of the data collection, with three cities chosen for study: Guangzhou, Foshan, and Shenzhen; **Guangzhou** (Canton), a rich metropolis bursting with vitality, it is the provincial capital and largest city of Guangdong Province, which borders the South China Sea on its eastern shore. Located on the Pearl River, which is accessible to the South China Sea, the city is Guangdong’s political, economic, scientific, educational, and cultural capital. A multifunctional metropolis since the Qin Dynasty (221–207 BC), Guangzhou has seen numerous transformations during the last two thousand years. Furthermore, it is one of China’s most famous tourist destinations. **Foshan** is a progressive city. Following China’s open-door policy, it was one of the first ports in China to engage in foreign trade. It has also grown to become the third-largest city in Guangdong Province due to manufacturing and tourism. In recent years, it has invested resources to further develop its thriving tourism business. It is located on the northern bank of the Pearl River, approximately 20 kilometers (12 miles) north of Guangzhou in Guangdong Province. The city, which dates all the way back to nearly 5,000 years ago, was named after three Buddha statues discovered in this location during the Tang Dynasty (618–907). **Shenzhen** is a young and modern metropolis in southern China adjacent to Hong Kong. It is best known for its fast development over the last decades; it attracts flowing business people to seek opportunities, especially cheap but good-qualified electronic products. It is also a great holiday destination with fabulous tourist attractions and markets for traditional local products.

A non-probability sampling method was used for the present study since it is difficult to obtain a representative sampling frame for social science studies or to find potential respondents from target populations to address research questions (Saunders et al., 2011). The researcher’s subjective judgment is the main consideration in non-probability sampling (Sekaran, 2006). A convenience non-probability sampling approach was used in this investigation. Two characteristics were used to collect data: (1) geography—that is, domestic and foreign respondents visiting the above-mentioned cities in Guangdong; selecting specific tourist attraction sites. (Guangzhou: Xiaozhou Village, Hakka village and Nangang Yao village – Foshan: Xiaqia Mountain, Ancient Nanfeng Kiln and Daqitou Village – Shenzhen: Dapeng village and Guru Local products market), and (2) time-based—that is, respondents selected during the National Day Festival which is also called GuoQingJie in 2021. It falls on October the 1st on the Gregorian calendar. The National Day is China’s second most important event after the Chinese New Year, when the majority of Chinese and foreign visitors participate in tourism activities. This facilitated data collecting and guaranteed that the population was more representative. A back-translation method was used to translate questionnaire items into Chinese. In order to ensure that the designed instrument is valid and usable, a pretest was conducted with 80 respondents to ensure there are no difficulties that may affect the quality of the gathered data. Based on Cochran’s Formula, a reasonable sample size of (584) respondents were determined (Wang J. et al., 2020). Hair et al. (2010) claim the margin of error is reasonable when the sample size is greater than 200. Some scholars, however, advise the use of structural equation modeling on sample sizes of at least 200 or between 10 and 20 cases per parameter (Kline, 2015). There were 900 returned questionnaires; of these, 644 usable questionnaires were retained after eliminating invalid and incomplete questionnaires, which exceeded the value of limited respondents.

### Data Analysis Procedures

The researchers used robust analysis to ensure the validity, reliability, and credibility of the findings using two statistically efficient software such as SPSS and SmartPLS 3.3.3. The descriptive analysis was done through SPSS. SmartPLS 3.3.3 was used during measurement modeling and structural modeling analyses (Henseler et al., 2014). For this purpose, first, we began our analyses by applying descriptive statistical analysis on participants’ demographics. Second, the measurement modeling analysis was performed by applying factor loading, Cronbach’s alpha, roh_A, composite reliability, convergent reliability, and discriminant validity. We also solved the collinearity problems, and improved model fit along with presenting the detail related to model explanatory power. Third, we also performed descriptive analysis on scales used in the research model to measure mean scores and standard deviation. Lastly, we applied the structural equation modeling technique through SmartPLS 3.3.3 to measure the direct and indirect relationships among constructs used in the research model.

### Demographics

Demographic data for the present study can be found in Table 2. Of the 644 respondents whose answers were used, around 40.4 percent of respondents were male, while 59.6 percent were female; 66 percent were between the ages of 18 and 30, 17 percent were between the ages of 31 and 45, followed by those under the age of 18 (12.8 percent), 3.7 percent were between the ages of 46 and 60, and three respondents were over the age of 61. The respondents’ educational attainment was as follows: 38 percent of respondents had earned a 4-year bachelor’s degree, 44.8 percent earned a three-year diploma, 5.8 percent and 5.6 percent, respectively, had earned a high school diploma or a master’s degree and above, and 5.8 percent had obtained a middle school diploma. Regarding income level, approximately 28.3% of the respondents reported earning 1701–3000 CNY (Chinese yuan) in monthly income, while 21.8% of respondents earned 3001–4500 CNY, 13.4% earned 4501–6000 CNY, 25% earned below 1700 CNY, and 11.5% of respondents earned more than 6001 CNY. Finally, concerning the number of the collected questionnaires forms each city, 222 questionnaires were in Guangzhou (34.4%), Foshan 203 (31.6%), and Shenzhen 219 (34%).

**TABLE 2:** Sample characteristic (*N* = 644).

Items	Characteristic	Frequency	Percentage (%)
Gender	Male	260	40.4
	Female	384	59.6
	Total	644	100
Age	Below 18	82	12.8
	18–30	425	66.0
	31–45	110	17.0
	46–60	24	3.7
	Above 61	3	0.5
	Total	644	100
Income level	Below 1700	161	25.0
	1701–3000	182	28.3
	3001–4500	141	21.8
	4501–6000	86	13.4
	Above 6001	74	11.5
	Total	644	100
Education level	Middle School	37	5.8
	High School	37	5.8
	Diploma	289	44.8
	Bachelor	245	38.0
	Masters and above	36	5.6
	Total	644	100
Cities	Guangzhou	222	34.4
	Foshan	203	31.6
	Shenzhen	219	34.0
	Total	644	100

### Measurement Model

The statistical software SmartPLS 3.3.3 was applied to determine the association among variables used in the research model via structural equation modeling. SmartPLS 3.3.3 was selected because of its statistical efficiency and low sensitivity to sample size than other software for covariance-based SEM (Hair et al., 2019). Covariance-based structural equation modeling (CB-SEM) was a popular approach to measure the complex relationships between variables used in the research model up to the last decade. Since 2010, the trend has changed; scholars use partial least squares structural equation modeling (PLS-SEM) significantly in social sciences (Hair et al., 2017; Avkiran et al., 2018). The PLS-SEM approach is preferable to CB-SEM because it allows measuring the structural model with numerous constructs, indicator variables, and structural paths without imposing distributional assumptions on the data. More crucially, PLS-SEM is a causal-predictive approach to SEM that emphasizes prediction in estimating statistical models, whose structures are designed to provide causal explanations (Sarstedt et al., 2017). As a result, this technique overcomes the apparent dichotomy between explanation as typically emphasized in academic research – and prediction, which is the basis for developing different implications (Hair et al., 2019). In addition, user-friendly software packages are available, such as SmartPLS (Ringle et al., 2015), which generally require little technical knowledge about the method.

This study aimed to ascertain the relationship between DIF, TS, and TT. Thus, we investigated the model’s constructs for validity and reliability prior to evaluating the expected influences. The constructs’ validity and reliability are summarized in Table 3. The dependability index of each item’s loading factors is more than the threshold value of 0.60 (Henseler et al., 2014). Likewise, additional reliability measures such as Cronbach’s alpha, composite reliability, and rho A all exceed the recommended value of 0.70 (Henseler et al., 2014). For the purpose of determining the convergent validity of reflective constructs, the AVE (average variance extracted) approach was used. Each construct had an AVE value greater than the normal value of 0.50 (Hair et al., 2019). The reliability and convergent validity of all reflective constructs included in the research model were assessed in this study. Each construct’s AVE square root was bigger than the variance shared by all constructs. As a result, the conditions for reliability and validity have been met.

**TABLE 3:** Discriminant validity.

Constructs	A	ALP	LCP	TT	TS
	0.803				
Access to local products (ALP)	0.617	0.805			
Local community participation (LCP)	0.555	0.431	0.774		
Tourist trust (TT)	0.637	0.627	0.518	0.723	
Tourist satisfaction (TS)	0.792	0.689	0.559	0.684	0.781

Henseler and Ringle questioned Fornell and Larcker’s method of determining discriminant validity, claiming that it cannot be relied upon to determine discriminant validity (Henseler et al., 2014). They advocated using a heterotrait-monotrait (HTMT) approach to assess discriminant validity in partial least squares standard error of the mean (SEM) (Hair et al., 2019). We used this method to assess discriminant validity, which is believed to be a more appropriate method. The HTMT method is defined as the correlation between constructs and the correlations within the same construct items. Henseler, Ringle, and Sarstedt argued that HTMT should have a standard value smaller than 0.90 (Hair et al., 2017). Thus, HTMT values greater than 0.90 imply a lack of discriminant validity for the construct. Each construct’s HTMT value is less than 0.90, as indicated in Table 3. As a result, the measure met discriminant validity standards.

When analyzing structural equation modeling, ensure that the issue of collinearity has been resolved. When the Variance Inflation Factor (VIF) exceeds 5, it indicates that there may be a collinearity issue between the dimensions (Hair et al., 2011). In this study, the VIF value of the structural equation modeling is less than 5, ranging between 1.473 and 3.379, showing no collinearity across the study dimensions. The metrics SRMR, NFI, and RMS theta are often employed in PLS-SEM to assess the overall model’s suitability. The SRMR value has a range of 0 to 1. When the SRMR is less than 0.08, the model is considered excellent (Hu and Bentler, 1998). The NFI value ranges from 0 to 1. The greater the value of NFI, the better the performance. When NFI exceeds 0.9, it implies that the model fits well (Bentler and Bonett, 1980). The RMS theta value should only be used to evaluate reflecting measurement models. A value of RMS theta less than 0.12 suggests that the model fits well (Henseler et al., 2014). The SRMR for model evaluation verification in this study is 0.057. Despite the fact that the NFI score of 0.832 is less than 0.9, the difference is not significant. The value of RMS theta is 0.123. Despite being greater than 0.12, it is also acceptable. As a result, the model used in this investigation was shown to be pretty well suited in general. Table 4 displays the collinearity analysis and model fit.

**TABLE 4:** Collinearity and model fits.

Dimensions	Tourist trust	Tourist satisfaction	Model fits	
Authenticity	2.884	1.936	SRMR	0.057
Access to local products	1.958	1.644	NFI	0.832
Local community participation	1.533	1.473	RMS- Theta	0.123
Tourist satisfaction	3.379			

The R2 value is considered when evaluating a model’s explanatory ability. The R2 value ranges from 0 to 1—the greater the explanatory power, the greater the value. When the R2 value is close to 0.50, the model has modest explanatory power. When the R2 value is close to 0.75, the model has a high degree of explanatory power. According to Table 5, perceived ease of use has a 54.4 percent explanatory power for tourist trust. The explanatory power of perceived usefulness and perceived ease of use to tourist satisfaction is 70.4 percent. As a result, the model in this study explains the latent variables extremely well and has a high level of explanatory power.

**TABLE 5:** R square and F square.

Constructs	*R* square	*R* square adjusted	TS- *f*^2^	TT- *f*^2^
Tourist trust (TT)	0.544	0.542		
Tourist satisfaction (TS)	0.704	0.703		0.019
Authenticity (A)			0.490	0.076
Access to local products (ALP)			0.191	0.034
Local community participation (LCP)			0.041	0.055

The explanatory impact value *f*^2^ is used to detect the effect of exogenous variables on endogenous variables. When 0.02 < *f*^2^ ≤ 0.15, it is a small effect. When 0.15 < *f*^2^ ≤ 0.35, it is a medium effect. Furthermore, when *f*^2^ > 0.35, it is a large effect. It can be seen from Table 5 that the explanatory effect value *f*^2^ of Authenticity to TS is 0.490. It displays a large-effect explanatory ability. Access to local products to TS is 0.191 indicating medium effects. Local community participation to TS is 0.041 indicating a smaller effect. Table 5 further demonstrates that the explanatory effect value *f*^2^ of Tourist Satisfaction to TT is 0.019, indicating a smaller effect. Authenticity to TT is 0.076. It displays a smaller-effect explanatory ability. Access to local products to TT is 0.034, indicating smaller effects. Local Community participation to TT is 0.055, which also showed a smaller effect.

### Redundancy Analysis

Redundancy analysis was run through blindfolding in PLS-SEM to measure the predictive criterion accuracy based on Stone– Geisser’s *Q*^2^ value (Asghar et al., 2022), which assesses the quality of the model. Q-square is reflecting the predictability of the endogenous constructs. A cross validity redundancy analysis yielded the value of *Q*^2^ (=1–SSE/SSO), greater than zero. Table 6 exhibits that the values are acceptable for endogenous constructs in PLS-SEM.

**TABLE 6:** Cross validity redundancy analysis.

Constructs	SSO	SSE	*Q*^2^ (=1-SSE/SSO)
TS	4501	2595.137	0.423
TT	3858	2791.303	0.276

### Structural Model

SmartPLS-SEM 3.3.3 was utilized in this investigation in order to ascertain the associations between variables in the research model (Hair et al., 2019). Partial least squares is a variance-based structural equation modeling (VB-SEM) technique that enables the measurement model to be evaluated concurrently. This method enables the validity and reliability of the scales employed in the study model to be determined. We evaluated the hypothesized links between the model’s constructs in the structural model. The six structures’ direct influences are listed in Table 7.

**TABLE 7:** Direct relation.

Direct relations	Coefficient	Mean	*SD*	T statistics	*P* values	Results
Local community participation – > Tourist trust	0.153	0.154	0.039	3.944	0.000	Accepted
Authenticity – > Tourist trust	0.160	0.157	0.050	3.198	0.001	Accepted
Access to local products – > Tourist trust	0.261	0.262	0.047	5.565	0.000	Accepted
Local community participation – > Tourist satisfaction	0.133	0.137	0.032	4.220	0.000	Accepted
Authenticity – > Tourist satisfaction	0.530	0.528	0.038	13.811	0.000	Accepted
Access to local products – > Tourist satisfaction	0.305	0.306	0.038	8.089	0.000	Accepted
Tourist satisfaction – > Tourist trust	0.292	0.294	0.056	5.217	0.000	Accepted

The three DIF constructs directly influence TT. Furthermore, LCP exhibits a significant positive connection with TT (β = 0.153, *p* < 0.05), indicating that hypothesis H1.1 is supported. Likewise, authenticity has a substantial positive influence on TT (β = 0.160, *p* < 0.05), supporting hypothesis H1.2. ALP has a beneficial effect on the TT (β = 0.261, *p* < 0.05), confirming hypothesis H1.3. LCP displays a substantial positive connection with TS (β = 0.133, *p* < 0.05), indicating that hypothesis H2.1 is supported. Furthermore, there is a substantial positive association between authenticity and TS (β = 0.530, *p* < 0.05), which supports hypothesis H2.2. ALP exhibits a substantial positive connection with TT (β = 0.305, *p* < 0.05), supporting hypothesis H2.3. Similarly, TS displays a substantial positive connection with TT (β = 0.292, *p* < 0.05), implying that hypothesis H3 is accepted.

### Intervening Effect

**FIGURE 2:**
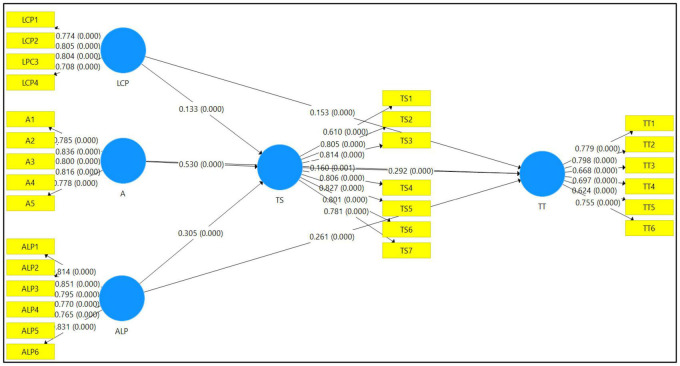
Structural equation model.

We tested the intervening effect of TS between DIF (LCP, A, ALP) and TT. As illustrated in Table 7, an indirect link was identified for LCP (β = 0.039, *p* < 0.05), A (β = 0.155, *p* < 0.05), and ALP (β = 0.089, *p* < 0.05) on TT. As a result, TS was demonstrated to have substantially and favorably mediated between LCP, A, and ALP, validating our hypotheses H4.1, H4.2, and H4.3 (see Table 8 and Figure 2).

**TABLE 8:** Indirect relation.

Coefficients	Coefficients	Means	*SD*	*T* statistics	*P*-values	Results
LCP – > TS – > TT	0.039	0.041	0.013	2.884	0.004	Accepted
A – > TS – > TT	0.155	0.155	0.032	4.845	0.000	Accepted
ALP – > TS – > TT	0.089	0.089	0.019	4.733	0.000	Accepted

## Discussion

The results derived from this study are meaningful because they were generated using a synthesized model framework. Only a few studies on rising countries like China have been published prior to this one. In addition, the few studies that have been done in emerging countries demonstrate a lack of focus on the tourism sector, despite its critical role in a country’s socioeconomic development. To the best of the author’s knowledge, this is one of the first studies investigating the impact of destination image formation on tourist trust in the Chinese heritage tourism context. The present study explores the direct relationship between DIF and TT. Additionally, we measured the direct relationship between DIF and TS. Likewise, we also measured the direct connection between tourist satisfaction and tourist trust. Moreover, the current research also measured the indirect relationship of destination image formation on tourist trust through TS in the Chinese tourism context.

We first focused on the direct relationship between DIF practices (LCP, A, ALP) and TT. The results revealed that all three dimensions of DIF pillars (LCP, A, ALP) significantly influence TT, which approved our hypotheses H1.1 to H1.3. Prior studies confirmed that DIF positively influenced on TT (Wang et al., 2021). Similarly, Huete-Alcocer et al. (2019) explored that DIF has been shown to be a significant predictor of positive TT. Additionally, the results from another study indicated that perceived authenticity is an essential element to build tourist trust in the tourism framework (Jeong and Kim, 2019b). It has also discussed the role of access to local products and building trust and personal relationships among different tourism stakeholders. Similarly, it was revealed that access to local products has a positive role in enhancing tourist trust (Roy et al., 2017). The plausible reason for this significant relationship was the quality DIF practices used to enhance the TT. Another reason might be that the three factors, such as LCP, authenticity, and ALP, worked effectively in the Chinese tourism industry, enhancing tourist trust.

Second, the study looked at the direct link between DIF (LCP, A, ALP) and TS. The results indicated that DIF (LCP, A, ALP) positively and directly affect TS, which approved hypothesis H2.1–H2.3. Prior studies confirmed the relationship between DIF (LCP, A, ALP) and TT (Jeong et al., 2019). Similarly, Chi and Qu (2008) found in their study that DIF has a positive and significant direct relationship with TS. The possible reason for this could be that the three factors such as LCP, A, ALP have delivered the relevant services in the most effective ways in the Chinese tourism industry, which enhance tourist satisfaction.

Third, the current study investigated the direct effect of TS on TT. The findings support hypothesis H3 by demonstrating that TS has a significant and positive effect on TT. Prior studies have also shown that TS positively influences TT. Chiu et al. (2016) and Lu et al. (2020) investigated the impact of cognitive image on TT among Chinese tourists visiting Korea. Therefore, it was deduced that the TS is a predictor of the TT. The tourist organizations must focus on maintaining the TS, which ultimately enhances the TT.

Finally, the study measured the indirect effect of DIF (LCP, A, ALP). The results confirmed that TS significantly and positively mediates between DIF (LCP, A, ALP) and TT, significantly supporting our hypotheses H4.1 to H4.3. Previous research has also shown that TS mediates between DIF and TT (Huyen and Thi Binh, 2020; Stylidis, 2020; Marine-Roig, 2021). Stavrianea and Kamenidou (2021) study also indicated that TS, DIF, and TT have a positive relationship with each other. dos Santos and Basso (2012) found that the facility of tourist-healthcare moderately impacts intent formation, while trust is developed in both personnel and the enterprise based on tourists’ satisfaction. Thus, it might be the cause of the (LCP, A, ALP) working more effectively with TS to enhance the TT. Both DIF and TS are predictors of TT.

## Conclusion

The results of this empirical study have valuable implications for managers and researchers. The present study’s findings enhance the discussion on jointly measuring the relationship among TS between DIF (LCP, A, ALP), TS, and TT. The results confirmed the relationships between DIF (LCP, A, ALP), TS, and TT, and TS also have mediating effects. We concluded the results of this study as follows: First, LCP plays an effective and positive role in building TT at destinations. Similarly, it was also considered that increased authenticity has a plosive and influential role in enhancing TT.

Additionally, ALP is also considered the predictor of TT. Second, LCP has an influential role in enhancing TS. Similarly, authenticity has an influential role in increasing the level of TS. Furthermore, ALP is also considered the predictor of TS. Third, TS has a positive role in enhancing the role of TT. Fourth, TS has an intervening role in strengthening the relationship between LCP and TT. Similarly, TS has an intervening role in strengthening the relationship between A, TT, and TS has an intervening role in strengthening the relationship between ALP and TT.

## Implications and Future Research Directions

### Practical/Managerial Implications

The image formation concept has received substantial focus in recent years as a key problem in the field of tourism research. However, many research gaps, especially the impact of tourist destination image formation (LCP, A, ALP), on tourist trust, and tourist satisfaction was used as a mediated variable were yet to be explored. Understanding these processes will have the benefit of providing destination marketers and managers with the knowledge they need to differentiate their destination offerings and foster a lifelong emotional connection with tourists. For this purpose, this study proposed a synthesized research model and evaluated it empirically. The results confirmed the strengthened proposed model, which is the first to include these factors and their relationships.

First and foremost, the findings of this study indicate that trustful visitors bring major advantages in terms of both competitiveness and economics. Key elements of the tourist connection, for instance, satisfaction and attachment, should be considered in addition to physical features by destination marketers or managers in order to better suit travelers’ actual and symbolic requirements. Destination managers’ concern for guests’ well-being and interests is shown in these characteristics. Tourist sites with a good reputation have a competitive advantage over others because of the trust that tourists have in it, such as whether or not their travel expectations are met or surpassed and how many related uncertainties are minimized, for example. An emotional connection can be formed between a place and its visitors if they have confidence in it, which can lead to increased return visits and recommendations from those who have been there. Therefore, destination marketers must rely on trust, developing a destination’s image and customer satisfaction to build long-term relationships with travelers that will lead to a loyal customer base.

Second, the current research demonstrates empirical evidence of relationships between DIF, TS, and TT. Previous scholars have asserted that destination image and TS are inextricably linked to trust (Lee et al., 2007; San Martin et al., 2013; Zhang et al., 2014; Wu, 2016). Others, though, disagree and have stated that tourist trust does not ensure visitors return to a site because they are typically predisposed to choose a new destination even when the experience is pleasant and happy (Trang, 2018). Moreover, Chi and Qu (2008) believe that destination trust has little to do with destination image. Nonetheless, the current study demonstrates and validates the presence of essential links between DIF, TS, and TT; the present study results provide valuable implications for researchers, managers, and supervisors. The tourist organizations should organize some professional training on improving their professional skills such as communication and management skills for their employees who are weak in these areas. The managers should establish the rewards and compensations for those employees. Moreover, customer satisfaction is also very important for building tourist trust; therefore, employees should be trained to enhance customer satisfaction at their workplaces. These steps can help to improve the destination’s image and increase tourist satisfaction.

Third, the current research contributes to tourist research by investigating the impact of DIF on TT while taking Tourist Satisfaction into account as a mediator. Prior research has consistently argued that in broad tourism settings, TS mediates the link between destination image and loyalty (Li et al., 2021), and our findings also identify an indirect relationship between DIF and destination trust via TS, which is consistent with past empirical findings or research (Jeong and Kim, 2019a). Particularly, the findings show that TS has a totally mediating influence, which has never been previously observed. Furthermore, based on the best available knowledge, the current study is the first in the context of Chinese tourism to include TS as a mediator of the relationship between DIF and tourist trust. The finding offers fresh insight on the entire mediatory function of TS in the relationship between (A, LCP, and ALP) and tourist trust among Chinese visitors. This evaluation is an important step toward a better understanding of TS in China.

In this respect, we contribute to the literature on tourism through our findings by proving the critical role of Destination Image formation (Authenticity, Access to local products, local community participation) and perceived value generated by it on TS and tourist Trust. We suggest that these variables should be considered when determining a tourism destination’s competitive advantage.

### Future Research Directions

The present study also has a few limitations that can affect the interpretation of its results. First, participants have come from a single country (China), which can create cultural biases and limit the generalizability of the results. More empirical pieces of evidence in other cultural contexts are required. We conducted this study to explore the impact of tourist DIF (A, LCP, ALP) on tourist trust, and tourist satisfaction was used as a mediated variable. Future studies should explore the mediating role of service quality, destination facilities, and employees behavior.

## Limitations

It cannot be denied that the current paper has several shortcomings that need to be addressed in future research. First and foremost, we only focus on tourist destinations in Guangzhou (Xiaozhou Village, Hakka Village, and Nangang Yao Village), Foshan (Xiaqia Mountain, Ancient Nanfeng Kiln and Daqitou Village), and Shenzhen (Dapeng village and Guru local products market), which are inside Guangdong Province, China. As a result, the context is limited to these areas and may differ in other locations. Moreover, surveys were conducted during COVID-19’s proactive measures, like quarantine and travel restrictions. Thus, longitudinal studies are necessary to achieve a better generalization of results.

Second, our study’s findings might not be relevant to other Asian tourist locations due to the fact that tourism features vary per country. Similar research in other tourist areas within China or in other countries with different cultures is necessary to generalize our findings. Additionally, this study is one of the few to inspect the effect of DIF on the TS and TT from the standpoint of a tourist’s decision-making process. The model should be replicated and validated in more areas to approve its use and validity.

Third, only three respondents over 61 were included in the study, which did not represent the overall Foreign and Chinese population, so it may not be possible to generalize the findings. This study had a relatively narrow scope and concentrated on a few key cultural heritage sites in Guangdong, China. As a result, the context is exclusive to this location and may differ in other locations. A sample size of greater than 30 but less than 500 is considered adequate for the majority of research (Jebbouri et al., 2021), and this study was successful in obtaining 644 respondents for the purpose of data analysis. A greater sample size, on the other hand, may result in a more normal distribution and hence more favorable results (Saunders et al., 2011). Last but not least, while this research collected data from participants using an on-site survey, different data gathering methods such as a self-administered questionnaire or interview might be employed to validate the research findings.

Finally, more research is required to examine the effects of additional variables so as to have a better comprehension of the dynamics that drive TS and TT. Finally, tourist satisfaction was investigated as a possible mediator of the relationships between DIF and tourist trust, as well as between TS and trust. Nonetheless, the impacts of additional possible mediators (for example, place attachment) ought to be researched in order to offer a more complete framework.

## Data Availability Statement

The raw data supporting the conclusions of this article will be made available by the authors, without undue reservation.

## Ethics Statement

Ethical review and approval was not required for the study on human participants in accordance with the local legislation and institutional requirements. Written informed consent for participation was not required for this study in accordance with the national legislation and the institutional requirements.

## Author Contributions

All authors listed have made a substantial, direct, and intellectual contribution to the work, and approved it for publication.

## Conflict of Interest

The authors declare that the research was conducted in the absence of any commercial or financial relationships that could be construed as a potential conflict of interest.

## Publisher’s Note

All claims expressed in this article are solely those of the authors and do not necessarily represent those of their affiliated organizations, or those of the publisher, the editors and the reviewers. Any product that may be evaluated in this article, or claim that may be made by its manufacturer, is not guaranteed or endorsed by the publisher.
