# Corrigendum: Impact of destination image formation on tourist trust: mediating role of tourist satisfaction

**DOI:** 10.3389/fpsyg.2023.1205955

**Published:** 2023-05-09

**Authors:** Abdelhamid Jebbouri, Heqing Zhang, Zahid Imran, Javed Iqbal, Nasser Bouchiba

**Affiliations:** ^1^Department of Tourism, School of Management, Guangzhou University, Guangzhou, China; ^2^Lahore Business School, University of Lahore, Lahore, Pakistan; ^3^School of Education, Guangzhou University, Guangzhou, China; ^4^Department of Political Science, Sun Yat-sen University, Guangzhou, China

**Keywords:** destination image formation, tourist satisfaction, tourist trust, authenticity, structural equation modeling, measurement analysis

In the published article, there was an error in the author affiliations as published. The affiliations were listed as “Abdelhamid Jebbouri^1†^, Heqing Zhang^2*†^, Zahid Imran^3†^, Javed Iqbal^4*†^ and Nasser Bouchiba^5†^” but should be “Abdelhamid Jebbouri^1†^, Heqing Zhang^1*†^, Zahid Imran^2†^, Javed Iqbal^3*†^ and Nasser Bouchiba^4†^.”

In addition, there was an error in affiliation 1 as published. Instead of “School of Tourism, Guangzhou University, Guangzhou, China” it should be “Department of Tourism, School of Management, Guangzhou University, Guangzhou, China.”

The authors apologize for these errors and state that this does not change the scientific conclusions of the article in any way. The original article has been updated.

